# Knowledge sharing of health technology among clinicians in integrated care system: The role of social networks

**DOI:** 10.3389/fpsyg.2022.926736

**Published:** 2022-09-27

**Authors:** Zhichao Zeng, Qingwen Deng, Wenbin Liu

**Affiliations:** Department of Health Management, School of Public Health, Fujian Medical University, Fuzhou, Fujian, China

**Keywords:** knowledge sharing, integrated care system, social network analysis, health technology, China

## Abstract

Promoting clinicians’ knowledge sharing of appropriate health technology within the integrated care system (ICS) is of great vitality in bridging the technological gap between member institutions. However, the role of social networks in knowledge sharing of health technology is still largely unknown. To address this issue, the study aims to clarify the influence of clinicians’ social networks on knowledge sharing of health technology within the ICS. A questionnaire survey was conducted among the clinicians in the Alliance of Liver Disease Specialists in Fujian Province, China. Social network analysis was conducted using NetDraw and UCINET, and the quadratic assignment procedure (QAP) multiple regression was used to analyze the influencing factors of knowledge sharing of health technology. The results showed that the ICS played an insufficient role in promoting overall knowledge sharing, especially inter-institutional knowledge sharing. Trust, emotional support, material support, and cognitive proximity positively influenced knowledge sharing of health technology, while the frequency of interaction and relationship importance had a negative impact on it. The finding extended the research scope of social network theory to the field of healthcare and will bridge the evidence gap in the influence of the clinicians’ social networks on their knowledge sharing within the ICS, providing new ideas to boost knowledge sharing and diffusion of appropriate health technology.

## Introduction

Known to us, knowledge has long been recognized as an important mean for organization to gain developmental resources and sustain competitive advantages (Goh and Sandhu, 2013; Wu et al., 2021). Knowledge sharing can be defined as the ongoing process of exchanging knowledge between individuals, groups, and organizations through knowledge exchange channels (Kipkosgei et al., 2020; Jiang and Chen, 2021). Since knowledge is often owned by an employee or a team, the success of knowledge management initiatives depends heavily on how effectively critical knowledge is being shared among employees. Additionally, prior studies also have extensively demonstrated that efficient knowledge sharing will greatly benefit the performance of certain organization (Tavakoli Taba et al., 2016; Lim et al., 2017; Butt et al., 2018). Thus, it is of vital importance to determine how to effectively promote knowledge sharing practice.

Healthcare organizations are a sort of knowledge-intensive organizations, which involves many clinicians with expertise in different specialities. Their continuous updating of knowledge and technology is the embodiment and requirement of professionalism, which is crucial for patient care, the quality of health services, and the reduction of medical errors (Gider et al., 2015; Yuan and Ma, 2022).By promoting the diffusion of health technology within and between organizations, knowledge sharing of appropriate health technology is commonly recognized as an efficient and effective way for health institutions to gain competitive advantages and improve performance (Jackson et al., 2006; Wu et al., 2021). And it also has been playing a crucial role in strengthening the capacity of health service delivery and subsequently benefiting the overall health of national citizens.

Factors that influence knowledge sharing include individual demographic attributes, interpersonal relationships, and personality traits. Demographic characteristics mainly include gender, social status, work department and workplace ownership attributes (Lee and Hong, 2014; Tausczik and Huang, 2020; Yuan and Ma, 2022). Studies on physician groups proved that differences in hospital ownership status, gender, age, position and departments led to barriers in knowledge sharing process, overload of work and negative attitude of senior physicians towards knowledge sharing (Huang, 2014; Gider et al., 2015). Besides different interpersonal relationship patterns (strong and weak ties) in social networks influence the extent of knowledge sharing (Levin and Cross, 2004; Naif Marouf, 2007; Huang, 2014; Hansen, 2016). In studies on social relationships of knowledge-intensive workers, weak relationships contributed to the learning and transfer of scientific methods and beliefs in different organizations. There is also evidence in studies that personality traits can contribute directly or indirectly to knowledge sharing, including altruism, conscientiousness, eagerness, and willingness (Obrenovic et al., 2020, 2021). In addition, the nature of knowledge (Brown et al., 2013), the relational dimension of social capital (i.e., trust) (Levin and Cross, 2004; Khvatova et al., 2016; Han et al., 2020), reciprocity (Zhang et al., 2021) and proximity (Broekel and Boschma, 2011; Zappa, 2011) were revealed to be important predictors of knowledge sharing.

However, even though the factors affecting knowledge sharing have been widely investigated, there are still some gaps existing in previous research on the aspects of objects, subjects and channels.

Firstly, there still exists a lack of an understanding of how to facilitate knowledge sharing in healthcare professionals, groups and organizations. At present, the main body of knowledge sharing research focuses on employees (Levin and Cross, 2004; Brown et al., 2013; Khvatova et al., 2016), students (Moghavvemi et al., 2017; Han et al., 2020), and so on. Comparing to these groups, the individuals and organizations in healthcare field are more knowledge-intensive. And the clinicians often have to leverage their expertise to collaborate with colleagues to solve the problems concertedly, which implies that they have a strong demand for knowledge sharing. Additionally, the output of medical care is health, which is not a tangible product as other industries (Chang et al., 2013). And the primary purpose of medical institutions is to heal the wounded and save the life, not to seek profit (Brennan et al., 2006). All these suggest that the knowledge sharing behavior in the medical field will be very different, which can be confirmed as a crucial issue for healthcare organizations and worth the effort required to conduct a comprehensive research.

Secondly, as for the object of knowledge sharing, there is also a dearth of study on the knowledge sharing of health technology, which is also a special kind of knowledge product. According to the classification of knowledge, many previous studies commonly divided the knowledge sharing into explicit and tacit knowledge sharing. Explicit knowledge sharing relies on facts, rules and policies that are communicated to potential recipients in written or electronic form (Wyatt, 2001; Jiang and Chen, 2021). Tacit knowledge sharing is not easy to directly codify in formal language because tacit knowledge is intuitive, subjective, and difficult to capture, and can only be achieved through frequent face-to-face interaction or observation (Huang et al., 2014; Obrenovic et al., 2021). However, the condition is much too complex for health technology to distinguish, especially for new health technologies. At its early diffusion stage with few technology adopters, the explicit knowledge sharing often dominates as exchanging the information of the new health technology by documents and meetings (Wiemken et al., 2012; Jiang and Chen, 2021). With the further diffusion and utilization of regarding technology, more tacit knowledge sharing tends to occur through interpersonal communication, interaction and cooperation, which not only simply completes the access and sharing of technology information, but also transforms and enriches the message and experience on technology utilization. This reminds us to pay attention to the subject of new health technology while investigating the knowledge sharing among healthcare professionals and organizations.

Thirdly, although the impact of interactive network on knowledge sharing has been investigated and confirmed in most previous studies (Brown et al., 2013; Huang, 2014; Khvatova et al., 2016; Han et al., 2020), little is known about the influence of social networks on knowledge sharing among individuals in different organizations. Social network theory generally starts from relational and structural elements, and focuses on variables such as the density, strength, scale of social connections, and the relative positions of network participants in the network to discuss the critical impact on knowledge and information flow. Especially under the context of widely implemented integrated care system (ICS) that integrates a variety of health institutions to provide seamless health services (Schussele Filliettaz et al., 2018; Nooteboom et al., 2019), some stable formal or informal social networks have been developed from long-term communication and cooperation between the member institutions of ICS while providing the wide spectrum of services with continuity. By taking advantage of these social networks that are often more or greater than those in fragmented health systems, the knowledge sharing of health technology within the ICS is expected to be more active and play more important roles in bridging technical gap between member institutions to improve the overall ICS service capacity. This also highlight the importance of corresponding research on knowledge sharing among clinicians, especially focusing on the influence of the social networks formed within the ICS on the clinicians’ knowledge sharing.

Fourthly, it is more appropriate to describe the interaction information of social network members by relational data. However, most studies apply the commonly used attribute data to measure the binary relationships, and conduct data analysis using logistic regression (Schussele Filliettaz et al., 2018), hierarchical linear models (Brown et al., 2013), structural equation models (Levin and Cross, 2004; Lin and Lo, 2015; Zhou, 2019), etc. It would lead to the deviation in research results for the ignorance of the intrinsic nature of knowledge sharing and the underlying interpersonal network.

Therefore, to bridge these knowledge gaps as mentioned above, this study will focus on the knowledge sharing in healthcare field, and aims to clarify the influence of clinicians’ social networks on knowledge sharing of health technology within the ICS by applying social network analysis. The findings will not only provide scientific evidence on the influencing factors underlying the knowledge sharing of social network members among different institutions, but also offer a practical basis for promoting the knowledge sharing and diffusion of appropriate health technology in the context of ICS.

The theoretical contributions and innovations of this study are as follows: Firstly, pay attention to the less focused area on knowledge sharing of health technology, which probably contain both explicit and tacit knowledge sharing during its technology diffusion process. Secondly, integrate social network into knowledge sharing in healthcare, and pay attention to both inter-organization and intra-organization knowledge sharing, which fill the knowledge gap of merely focusing on intra-organization knowledge sharing. Thirdly, use relational data and conduct social network analysis to describe and analyze the interaction information of social network members, which make the result more reliable and robust.

## Theory and model

### Theoretical framework

**Figure 1:**
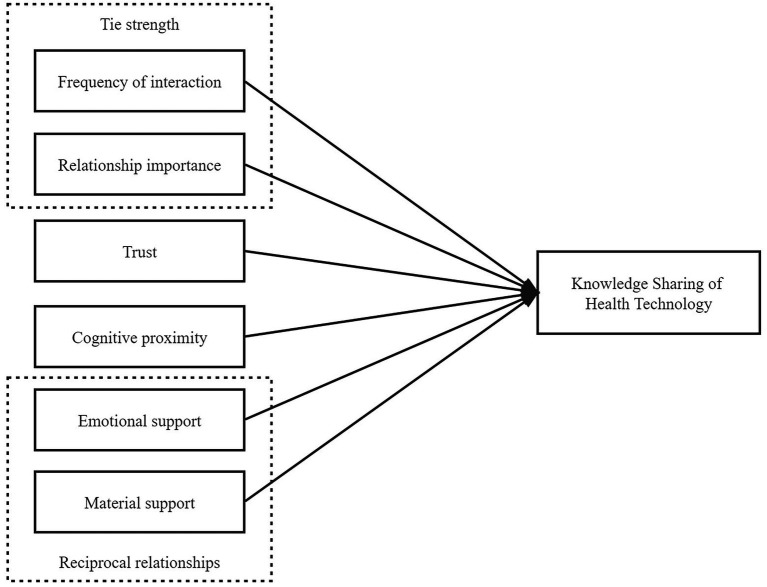
Theoretical framework of knowledge sharing of health technology.

Social network theory holds that the world is composed of relationships, and the relationships of social individuals are the channels for the flow of resources and information, thus constituting the multi-layered nature of social networks. The weak ties theory, structural hole theory, embeddedness theory, and six degrees of separation theory are the most representative ones. Weak ties theory indicates that differences in individuals bring heterogeneous information and become a bridge for communication across different groups, contributing to the dissemination and diffusion of explicit knowledge (Liu and Duff, 1972; Granovetter, 1973; Cai and Abouzahra, 2022). Strong ties are links that maintain relationships within an organization, leading to duplication of resources and information. But some other scholars have questioned the weak ties theory. Bian acknowledged that weak ties played a crucial role in information transfer, and also suggested that strong ties based on trust and obligation were more advantageous (Bian and Ang, 1997). Burt also found that having duplicate information and disconnected structural holes was a prerequisite for building information bridges, suggesting that there was no essential difference between strong and weak ties (Burt, 2004, 2012). Next, embeddedness theory considers that economic behaviors and outcomes are affected by the ties between actors and the structure of relational network, including relational embeddedness, structural embeddedness, and cognitive embeddedness (Boschma et al., 2002; Granovetter, 2018). Relational embeddedness focuses on binary issues such as reciprocity, trust, and collaboration. Structural embeddedness includes strong and weak ties, network centrality, network centralization, etc. Cognitive embeddedness believes that the knowledge structure and attitudes can determine knowledge transfer (Dequech, 2003). Thus, based on social network theory and previous researches, this study hypothesized that frequency of interaction, relationship importance, trust, cognitive proximity, material support, and emotional support had a significant impact on the clinicians’ knowledge sharing of health technology within the ICS. The theoretical framework was presented as follows (Figure 1).

### Research hypotheses

#### Tie strength

Tie strength refers to the closeness of the social relationship between clinicians, including the frequency of interaction and relationship importance (Huang, 2014). Tie strength can be defined as a combination of the amount of time, emotional intensity, intimacy, and reciprocity (Granovetter, 1973), so frequency of interaction and relationship importance were used to represent tie strength in the study. Frequency of interaction refers to the number of times an individual communicates with each other within a certain period, including face-to-face or telephone communication. A study of virtual teams found frequent interaction was pivotal for successful knowledge exchange (Tietz et al., 2021). Other studies showed that frequent communication online or offline was positively correlated with tie strength, promoting knowledge dissemination (Kim and Fernandez, 2017; Dissing et al., 2018). Ongoing interaction among partners provides for knowledge exchange, which builds a common system of knowledge within an organization. Relationship importance leads individuals to interact frequently with few relatives or friends and less often with a large number of acquaintances. It will affect the frequency of communication between knowledge sharing actors, which in turn affects knowledge dissemination. Various studies on social relationships of knowledge-intensive employee have implied that strong ties were more efficient at conveying complex information within an organization (Reagans and Zuckerman, 2001; Chung and Hossain, 2009). Within ICS, knowledge sharing of health technologies requires a top-down transfer of skills and experience, which is relatively easy to achieve and maintain when there is a strong interaction between individuals. Based on the above considerations, the following assumptions are made:

H1a: Frequency of interaction has a positive effect on knowledge sharing of health technology.

H1b: Relationship importance has a positive effect on knowledge sharing of health technology.

#### Trust

Mayer et al., (1995) have defined trust as “readiness to accept the influence of another party’s actions based on the belief that the other party will accomplish a particular task relevant to the trustor, irrespective of the ability to monitor the other party.” Interpersonal trust is a precondition for building knowledge sharing (Ismail and Yusof, 2010; Smaliukienė et al., 2017), guiding social interactions between individuals, stimulating the organization’s contributions and existing resources, and thus enhancing the organization’s innovation (Ouakouak and Ouedraogo, 2018). Several studies supported the impact of trust on knowledge sharing. A study on a virtual team identified trust as an effective way for employees to open up and analyze information, especially unable to meet face-to-face (Pinjani and Palvia, 2013). When trust is present, employees are willing to listen to each other and are more likely to realize the value of new knowledge and apply it, thereby facilitating knowledge transfer and communication (Kipkosgei et al., 2020). Chen et al. demonstrated that the presence of high trust between individuals can push people to participate more actively in social communication and cooperation (Chen and Huang, 2007). Based on this, the following hypothesis are proposed:

H2: Trust has a positive effect on knowledge sharing of health technology.

#### Cognitive proximity

Cognitive proximity is the similarity between individuals in the construction of knowledge systems in a certain field, thereby affecting attitudes and willingness to use health technologies (Criscuolo et al., 2010; Broekel and Boschma, 2011). It determines that individuals have different perceptions and attitudes of the interpretation of the world, and these perceptions are the basis for action. The importance of cognitive proximity to knowledge dissemination is self-evident and is considered as a prerequisite for knowledge transfer and interaction. Studies have shown that cognitive proximity may have both positive and negative effects on knowledge transmission. Some studies believed that knowledge creation required the integration and complementation of multiple heterogeneous cultures. Too similar cognition will result in the convergence of thinking and knowledge of organizational members, thereby hindering the input of heterogeneous cultures within the organization and reducing the possibility of knowledge innovation and integration (Broekel and Boschma, 2011; Parjanen et al., 2011; Nieves and Osorio, 2015). Contrary to the above arguments, other studies have suggested that the higher degree of similarity in knowledge systems among members, the more conducive to knowledge exchange and cooperation, especially in highly specialized fields (Choi and Thompson, 2005; Criscuolo et al., 2010; Marrocu et al., 2013). Physicians, as knowledge-intensive talents, necessitate to constantly update their skills and knowledge due to professionalism. The homogenization of knowledge can effectively reduce the barriers to communication and understanding, and promote the reorganization of medical knowledge and the refinement of appropriate technologies. Based on the above, this study proposes the following hypothesis:

H3: Cognitive proximity has a positive effect on knowledge sharing of health technology.

#### Reciprocal relationships

Reciprocal relationships, including material support and emotional support in this study, enable knowledge sharing behavior to occur over time (Goh and Sandhu, 2013), and can be defined as the extent to which mutually beneficial cooperative relationships are established with other actors (Zhang et al., 2017). Reciprocity is the basic principle of social interaction, emphasizing the active and voluntary sharing of knowledge by individuals. If the material reward is insufficient, the individual will refuse to transfer knowledge outward. Research in sport organizations found that organizational rewards and enjoyment of helping others were positively related to knowledge sharing attitudes and behaviors (Jaberi et al., 2013), and study of nursing staff (Rafieian-Isfahani et al., 2020)and tourism industry staff (Fung and Hon, 2021)also yielded consistent results. Emotional support refers to individuals’ participation in social communication in order to obtain a valuable resource or benefit (Ellison et al., 2007), such as reputation, social recognition. Hsu and Lin concluded that people hoped to improve their social image by contributing knowledge (Hsu and Lin, 2008). Other studies suggested that social recognition of knowledge disseminators by others will promote the generation of individual knowledge sharing behaviors (Hossain et al., 2018; Choi et al., 2020). Therefore, material support and spiritual support can encourage individuals to actively share ideas. Based on this, we hypothesize that:

H4a: Material support has a positive effect on knowledge sharing of health technology.

H4b: Emotional support has a positive effect on knowledge sharing of health technology.

## Materials and methods

### Study setting

As a high-incidence area of liver cancer, Fujian Province is faced with the high mortality rate all year round (Zhou et al., 2021). However, some confirmed appropriate and effective liver cancer screening technologies having been routinely used in large medical institutions, have still not been commonly applied in primary health care (PHC) institutions (Deng et al., 2021a,b). To make this study more focused and feasible, the liver disease specialist medical alliance in Fujian Province of China was selected as a research case. This medical alliance was established in 2016, and consisted of a regional high-level hepatobiliary tertiary hospital and 20 county hospitals from 9 cities in Fujian Province. With the tertiary hospital as the leading institution at the core, all the member institutions of this medical alliance conducted mutually beneficial cooperation in terms of technical support, staff training, and patient referral.

### Measures

According to the theoretical framework proposed in Figure 1, the questionnaire for the knowledge sharing of health technology was developed consisting of two parts (see Supplementary material). The first part was the participant’s social network information. This part adopted the nomination method adapted by Valente and colleagues (Valente and Pumpuang, 2007). Each participant was required to self-report 1–3 clinicians in the ICS with which he/she share knowledge of health technology and provide the direction of knowledge sharing, including (1) He/she share knowledge to me (2) I share knowledge to him/her (3) We share knowledge with each other. In addition, participants were asked to offer other information about the interactions with each nominee, including emotional support and its direction, material support and its direction, trust, frequency of interaction, importance of relationship and cognitive proximity. Since similar individual demographic attributes increase the possibility of interaction (McPherson et al., 2001; O'Malley and Marsden, 2008; Borgatti et al., 2018), to control its potential impact, the second part of the questionnaire collected information on gender, professional title, and department. The assignment of all variables was shown in Table 1.

**Table 1:** Assignments of variable.

Variable	Assignments
Knowledge sharing of health technology	0 (No mention), 1 (knowledge sharing of health technology)
Gender	0 (Different gender), 1 (the same gender)
Professional title	0 (Different professional title), 1 (the same professional title)
Department	0 (Different department), 1 (the same department)
Emotional support	0 (No mention), 1 (emotional support)
Material support	0 (No mention), 1 (material support)
Trust	0 (No trust), 1 (less trust), 2 (moderate trust), 3 (very trust), and 4 (absolutely trust)
Frequency of interaction	1 (Once a year or less), 2 (2–5 times a year), 3 (6–11 times a year), 4 (1–3 times a month), and 5 (once a week or more)
Relationship importance	1 (Not important), 2 (generally important), 3 (important), 4 (very important), and 5 (absolutely important)
Cognitive proximity	0 (Different), 1 (somewhat similar), 2 (nearly the same), 3 (almost the same)

#### Dependent variable

*Knowledge sharing of health technology.* Knowledge sharing of health technology is a dependent variable network in this study. To obtain the network of knowledge sharing of health technology, we used the following questions, ‘What is the direction of knowledge sharing of health technology between you and him/her?’ Each of the 123 respondents in the study had the potential to establish knowledge sharing links with 122 other clinicians, yielding 15,006 observations. We created a 123 × 123 knowledge sharing matrix using the name generator term for the nominated knowledge sharing object, where a “1” in cell ij indicates that i mentions the existence of knowledge sharing with j and a “0” indicates no mention.

#### Independent variables

##### Emotional support

The network of emotional support was tested by extracting the corresponding question by McAllister (McAllister, 1995; Liou et al., 2016; Permwonguswa et al., 2018). We asked, ‘When you encounter difficulties at work (such as doctor-patient conflicts, etc.) or feel uncomfortable, and need emotional and psychological comfort, what is the direction of emotional support between you and him/her?’ We created a 123 × 123 emotional support matrix from the information filled in by the respondents about their emotional support with the nominee, where a “1” in cell ij indicates that i mentions the presence of emotional support for j and a “0” indicates no mention.

##### Material support

To measure the network of emotional support (Bock and Kim, 2005; Lee et al., 2021), we asked, ‘When you need to use things you do not have (such as medical equipment, presentation PPT, etc.) because of your work, what is the direction of your material support behavior with him/her?’ We created a 123 × 123 material support matrix from the information filled in by the respondents about their material support with the nominee, where a “1” in cell ij indicates that i mentions the presence of material support for j and a “0” indicates no mention.

##### Trust

Since trust is related to length of acquaintance (Singh and Srivastava, 2009; Khvatova et al., 2016), we use length of acquaintance to measure the level of trust (i.e., the longer length of acquaintance, the higher the trust level). For length of acquaintance the question was, ‘How long have you known him/her?’ We created a 123 × 123 trust matrix from the length of acquaintance filled in by the interviewee with the nominee. The values of cell ij of the length of acquaintance matrix ranged from 0 (means that no contact exists between the two clinicians) to 4 (means that the two clinicians have acquainted more than 10 years).

##### Frequency of interaction

The network of frequency of interaction was measured with reference to a scale developed by Wilson (Wilson, 1988).We asked, ‘In the past year, how often did you interact with him/her?’ We created a 123 × 123 frequency of interaction matrix from the frequency of interaction filled in by the interviewee with the nominee. The values of cell ij of the frequency of interaction matrix ranged from 0 (means that no contact exists between the two clinicians) to 5 (means that the frequency of interaction of two clinicians are once a week or more).

##### Relationship importance

To measurement of the network of importance of relationship, we referred to the scale by Chung et al. (Chung and Hossain, 2009). We asked, ‘What do you think is the importance of the relationship with him/her?’ We created a 123 × 123 importance of relationship matrix from relationship importance filled in by the interviewee with the nominee. The values of cell ij of relationship importance matrix ranged from 0 (means that no contact exists between the two clinicians) to 5 (means that the relationship between the two clinicians is absolutely important).

##### Cognitive proximity

For cognitive proximity, we measured an item selected from the scale developed by Butler (Butler and Cantrell, 2016). The question was, ‘The degree of your and his/her cognition in health technology.’ We created a 123 × 123 cognitive proximity matrix from filled in by the interviewee with the nominee. The values of cell ij of cognitive proximity matrix ranged from 0 (means that the two clinicians’ cognitions about health technology are different) to 3 (means that the two clinicians have almost the same cognition about health technology).

#### Control variables

Based on previous studies (Kim et al., 2016; Borgatti et al., 2018), we used three variables (same gender, same professional title and same department) to control for the effect of homogeneity (similarity in participants’ backgrounds increased their chances of interacting). In the gender matrix, cell ij was 1 if clinician i and clinician j had the same gender. In the professional title matrix, cell ij was 1 if clinician i and clinician j had the same professional title. In the department matrix, cell ij was 1 if clinician i and clinician j had the same department.

### Data collection

To investigate how social networks affect the clinicians’ knowledge sharing of health technology within the ICS, the liver disease-related unit of a liver disease specialist medical alliance participated in the study, including hepatology, oncology, gastroenterology, infection, ultrasound, etc. The survey was conducted during April and June 2019.

We conducted an on-site survey of all institutional members of the liver disease specialist medical alliance. The distribution of the questionnaires was accompanied by a trained coordinator to introduce the study purpose and the use of data. After fully understanding what they would need to do and how to do it, the clinicians were asked to complete an informed consent form if they agreed to participate in the survey. Notably, the participants were informed that they always had the right to immediately withdraw from the study at any time without any reason, and their interests will not be harmed by dropping out of this study. In the part of social network information, participants were asked to indicate 1–3 clinicians that perform knowledge sharing of health technology and provide social interaction information including emotional support, material support, trust, frequency of interaction, relationship importance, and cognitive proximity. From the participants’ self-reports, ten social matrices represented at knowledge sharing of health technology, emotional support, material support, trust, frequency of interaction, relationship importance, cognitive proximity, gender, professional title and department were determined. The relationships captured in the preceding seven matrices are all asymmetric, which means that the captured relationships are directional rather than reciprocal. In order to conduct the network matching, participants were asked to use the real names of the nominees and themselves in the nomination process. Following standard procedures for social network research, the results were anonymized to protect privacy (i.e., numbers were used instead of people’s names) (Paxton et al., 1999; Hutchinson and Rapee, 2007; Bruening et al., 2012; Shin et al., 2014; Cockerham et al., 2017).

### Evaluation index of network

Density and degree centrality are important indexes for the evaluation of social networks (Freeman, 1978; Provan and Sebastian, 1998). Network density is an index that describes the closeness of the relationship between actors, which is defined as the ratio of the number of existing relationships or ties to the number of all potential ties (Naif Marouf, 2007; O'Malley and Marsden, 2008). If the density exceeds 0.3, it can be considered as high density (Everett and Borgatti, 2005). Degree centrality measures the number of connections or contacts maintained by each respondent, which reflects the extent to which network members participate in the relationships within the network (Naif Marouf, 2007).

### Data analysis

EpiData (version 3.1) was used for data entry and exported to Microsoft Excel. The data were expressed in the form of adjacency matrix, with each participant as one node in the network. According to participants’ self-reported knowledge sharing of health technology and its direction, the matrix of knowledge sharing of health technology was identified. And the matrix of the other variables, such as emotional support, material support, trust, importance of relationship, cognitive proximity, gender, professional title, and department, were also determined on the basis of the information about interactions and individual demographic attributes. Then, the data of adjacency matrix were entered into UCINET (version 6) and NetDraw (version 2) to do social network analysis, including network visualization to show the scale and structure of the network clearly, and evaluation of density and degree centrality of the network (Hazrati et al., 2021; Yao et al., 2022; Zeng and Li, 2022).

The quadratic assignment procedure (QAP) multiple regression test in UCINET was used to examine the theoretical model. Since QAP regression overcomes the inherent error autocorrelation problem in network data (Krackhardt, 1988; Dekker et al., 2007), it is usually applied to the regression analysis of network data. QAP accounts for the non-independence of network data in two steps. Firstly, ordinary least squares (OLS) regression coefficients are calculated in the usual way. Second, a null hypothesis reference distribution of the regression coefficients and *R*^2^ values is generated and compared to the observed coefficients (from step one) to determine their statistical significance. To create this reference distribution, QAP randomly aligns (i.e., rearranges) all rows and matching columns of the dependency matrix and recalculates the regression for the resulting alignment matrix. This step is repeated 2000 times to estimate the reference distribution of regression coefficients and *R*^2^ values.

QAP regression was conducted in this study using knowledge sharing of health technology as the dependent variable, individual demographic attributes (gender, professional title, and department) as control variables, and emotional support, material support, trust, frequency of interaction, relationship importance, and cognitive proximity as independent variables. Network logistic regression for hypothesis testing was conducted with 5,000 times iterations. The criterion of significance was set to be 0.05 for this test.

## Results

### Descriptive statistics of the network of knowledge sharing of health technology

In the surveyed knowledge sharing network of this liver disease specialist medical alliance, the number of possible connections is 15,006 [= n* (n-1)]. The actual number of relations is 191, so the density of the knowledge sharing network in this study was 0.013, which meant that only 1.3% of all possible knowledge sharing of health technology was actual. The distribution of degree centrality was shown in Table 2. The clinicians’ degree centrality averaged 1.984, and the values of degree centrality for the overwhelming majority of clinicians (96.8%) ranged from 1 to 4.

**Table 2:** The distribution of degree centrality.

Degree centrality	Frequency (*n*)	Percentage (%)
1	58	47.2
2	31	25.2
3	20	16.3
4	10	8.1
5	2	1.6
6	1	0.8
8	1	0.8

### Results of network visualization

**Figure 2:**
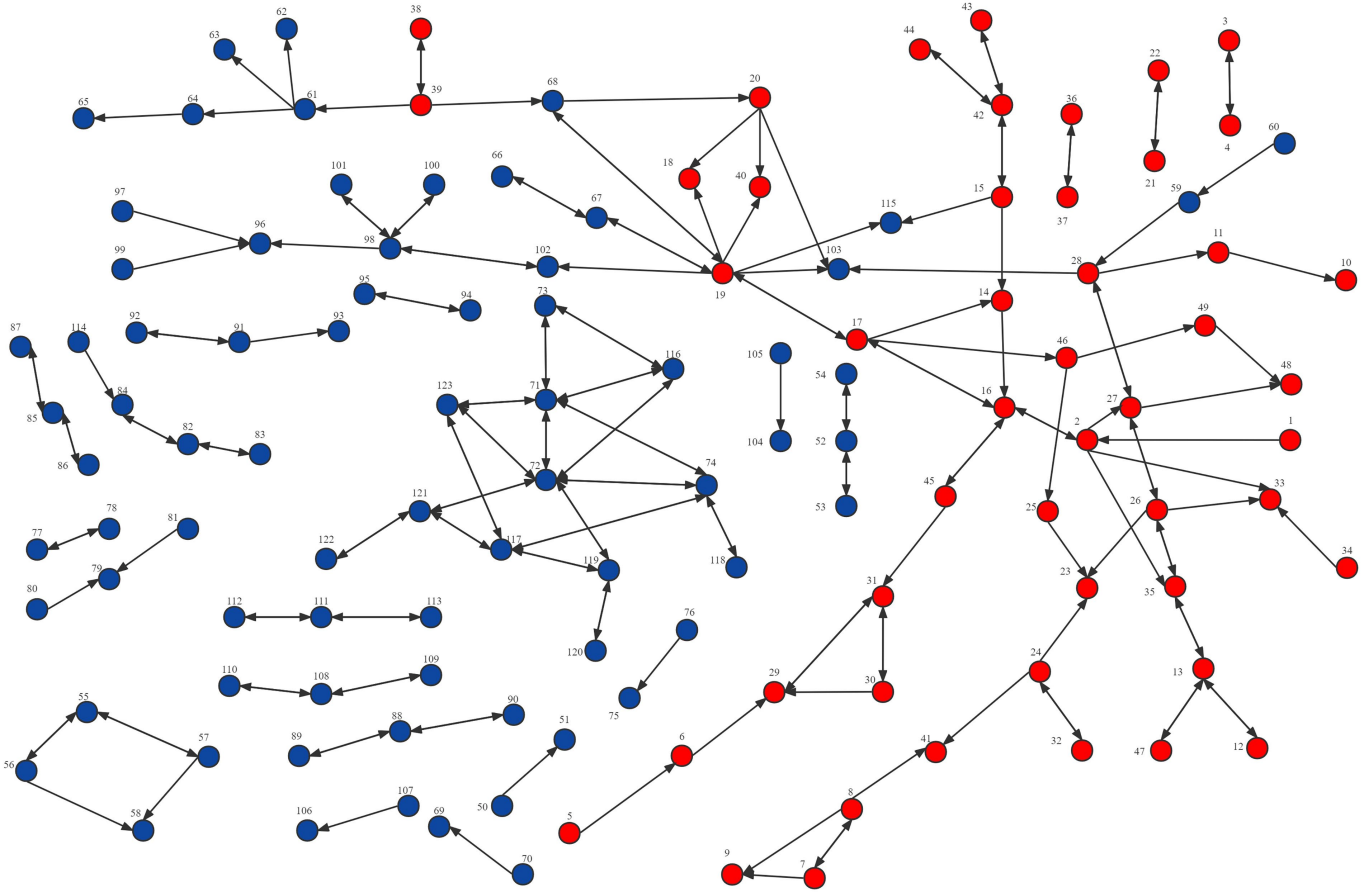
Network visualization of the overall knowledge sharing network for health technology in the Fujian liver disease specialist medical alliance.

**Figure 3:**
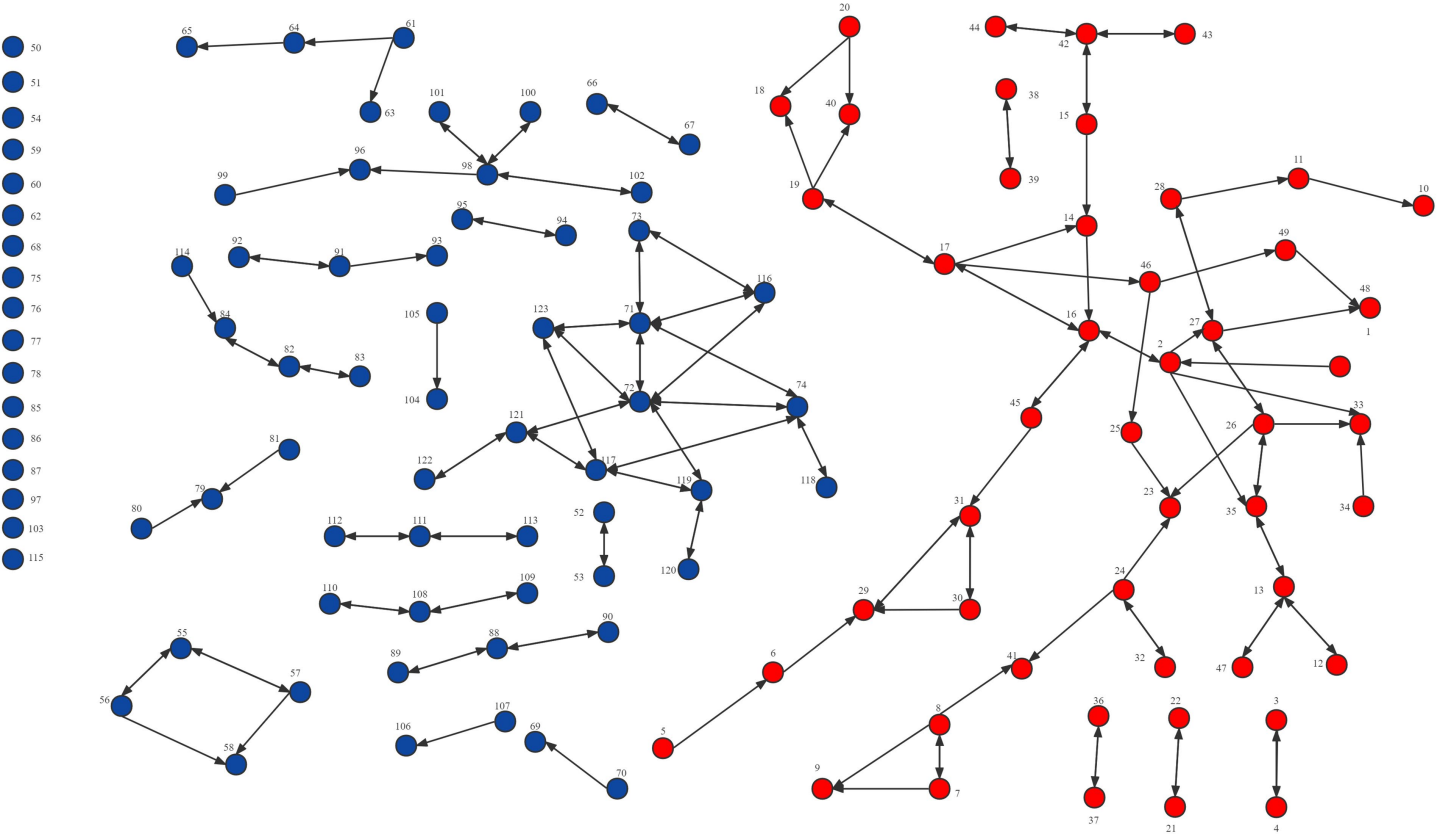
Network visualization of the intra-organizational knowledge sharing network for health technology in the Fujian liver disease specialist medical alliance.

**Figure 4:**
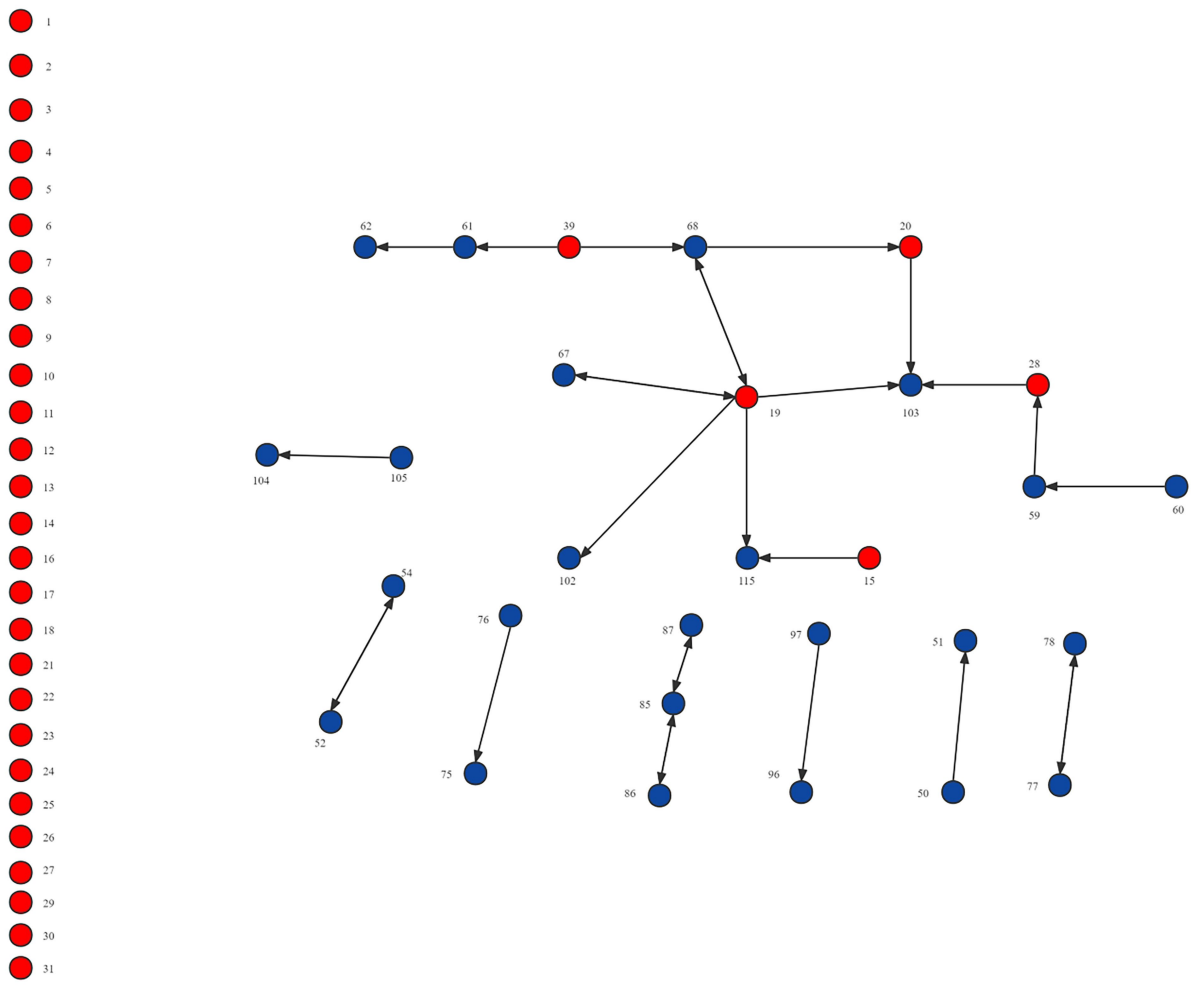
Network visualization of the inter-organizational knowledge sharing network for health technology in the Fujian liver disease specialist medical alliance.

Network visualization is another useful analysis method that can reveal structural patterns such as the number of nodes and relationships and network structure. Figures 2–4 demonstrated the map of the overall, intra-organizational, inter-organizational knowledge sharing network for health technology, respectively. There are 123 nodes in each figure, each node represented a clinician, and the red nodes represented clinicians from the leading institution, while the blue nodes represented clinicians from non-leading institutions. Arrows and links indicated the direction of knowledge sharing among clinicians. There were 191 links and no isolated points in the overall knowledge sharing network for health technology. The number of links for the intra-organizational (density = 0.077) and inter-organizational (density = 0.002) knowledge sharing network for health technology was 161 (84.3%) and 30 (15.7%), respectively.

### Results of QAP multiple regression

The results of QAP multiple regressions were demonstrated in Table 3. Two models were constructed in this study. Model 1 only contained control variables, and model 2 contained all variables including control variables and independent variables. In model 1, the *R*^2^ value for model 1 was 0.010, indicating that the three independent variables together explain 10.0% of the variance in knowledge sharing of health technology. Department had a positive impact on knowledge sharing of health technology (*B* = 0.102, *p* < 0.001), but the influence of gender (*B* = 0.009, *p* = 0.197) and professional title (*B* = 0.007, *p* = 0.262) were not significant. In model 2, the *R*^2^ value for model 2 was 0.896, indicating that the nine independent variables together explain 89.6% of the variance in knowledge sharing of health technology. In previous QAP regression analysis on knowledge sharing, the *R*^2^ values ranged from 0.560 to 0.718. The *R*^2^ value for model 2 was excellent, and the index was good. Therefore, it had good explanatory power and was accepted as the final model. Emotional support (*B* = 0.349, *p* < 0.001), material support (*B* = 0.584, *p* < 0.001), trust (*B* = 0.082, *p* < 0.001), and cognitive proximity (*B* = 0.041, *p* = 0.001) had positive impacts on knowledge sharing of health technology, while frequency of interaction (*B* = -0.053, *p* < 0.001) and relationship importance (*B* = -0.035, *p* = 0.019) had negative impacts. All control variables (gender, professional title, and department) had no significant impact.

**Table 3:** Result of QAP multiple regression.

	Model 1		Model 2	
	*B*(exp(b))	*P*	*B*(exp(b))	*P*
Gender	0.009	0.197	0.003	0.124
Professional title	0.007	0.262	0.002	0.214
Department	0.102	<0.001	−0.001	0.072
Emotional support	–	–	0.349	<0.001
Material support	–	–	0.584	<0.001
Trust	–	–	0.082	<0.001
Frequency of interaction	–	–	−0.053	<0.001
Relationship importance	–	–	−0.035	0.019
Cognitive proximity	–	–	0.041	0.002
Adj *R*^2^	0.010		0.896	

## Discussion

Guided by social network theory, this study investigated clinicians’ knowledge sharing of health technology. Current empirical research had paid much attention to the influence of social networks on intra-organizational knowledge sharing (Brown et al., 2013; Khvatova et al., 2016; Han et al., 2020; Lee et al., 2021), and improperly applied the commonly used attribute data to measure the binary relationships. This study turned the focus on knowledge sharing among individuals in different organizations, and appropriately described the interaction information of social network members by relational data. It applied NetDraw and UCINET to draw network maps and evaluate network structure (network density, degree centrality, etc.) of clinicians’ knowledge sharing of health technology within the ICS, and conducted QAP analysis to identify its regarding influencing factors. The findings of this study will not only bridge the evidence gap in the influence of the clinicians’ social networks on their knowledge sharing within the ICS, but also provide new ideas to promote knowledge sharing and diffusion of appropriate health technology.

An analysis of the density, degree centrality, and network visualization of the network of knowledge sharing of health technology demonstrated that the inter-organizational network density (0.002) was lower than the intra-organizational network density (0.077), which revealed that most clinicians have much less knowledge sharing with ones at the member institutions than those in the same institution. It implies that even if a medical alliance is established, its role of promoting technology sharing among member institutions is still open to question. This might be related to the characteristics of collaborative model within ICS, which had only limited collaboration in technical support, staff training, financial support, and patient referral, and no clear rights and obligations between member institutions. The loose cooperation model made it difficult for the ICS to form a community of interest, which in turn led to a lack of motivation among member institutions to promote knowledge sharing. Additionally, the positive impact of geographical proximity for knowledge sharing was confirmed in previous studies (Broekel and Boschma, 2011; Zappa, 2011). The medical alliance member institutions are distributed in 9 different cities in a province, making it tricky to sustain face-to-face knowledge sharing, which may also increase its communication costs, reduce the efficiency of information exchange, fail to retain information, and ultimately hinder knowledge sharing within ICS. Therefore, social media or online communities can be introduced to facilitate the dissemination of new knowledge and technologies within ICS. Social media has been identified as a critical driver of knowledge sharing (Edo-Osagie et al., 2020; Yao et al., 2022), providing a constant and easily accessible platform where members can break down geographic barriers and communicate freely with knowledge senders (Zhang et al., 2018; Ainley et al., 2021).

Consistent with previous fundings, this study also confirmed that trust, cognitive proximity, and reciprocal relationships (material support and emotional support) had a significant positive effect on knowledge sharing of health technology. Trust is the prerequisite for the occurrence of knowledge sharing (Levin and Cross, 2004; Zhou, 2019), which can maintain communication relationships and in turn prompt the quantity and quality of knowledge sharing. And, mutual trust is particularly considerable to create a knowledge-sharing atmosphere. Especially, in the highly competitive medical industry environment, clinicians are reluctant to share, and the purpose can only be achieved by promoting the degree of trust among physicians. Because trust is an interactive process, individuals can get satisfaction from each other (Kipkosgei et al., 2020). Previous studies had different views on cognitive proximity. Some studies suggested that a high degree of cognitive proximity may lead individuals to confront the risk of weakening competitiveness or to obtain less benefit from cooperation in innovative activities. But the reason for the positive effect of it could be inferred from studies indicating the conducive role of the common awareness and understanding of health technology in reducing the cost (Criscuolo et al., 2010). Notably, the medical field is a highly specialized industry and should always keep a cautious and meticulous learning attitude. When cognitions are inconsistent, there may be a problem of inability to absorb knowledge. Concerning the reciprocal relationships, its positive role in enhancing mutual trust and working together to overcome difficulties would contribute to the smooth advancement of knowledge sharing (Dahlander and McFarland, 2013).In addition, the knowledge sender expects reciprocal relationship as proof of the time and effort invested in the knowledge sharing process, and expects to get help from others when needed, which will also foster knowledge sharing (Zhang et al., 2021).

It is noteworthy that this study demonstrated the dominant role of weak ties in knowledge sharing of health technology, as tie strength (frequency of interaction and relationship importance) had a significant negative impact on regarding knowledge sharing. As shown in previous studies, weak ties are related to explicit knowledge sharing as an effective channel for transferring information between different social clusters, while strong ties are associated with tacit knowledge sharing (Huang, 2014; Hansen, 2016). The results revealed that the knowledge shared by clinicians within the ICS was mainly explicit knowledge, rather than the tacit knowledge of health technology. There were two plausible reasons for this phenomenon. On the one hand, it might result from the characteristics of knowledge sharing in the early stage of health technology diffusion (Brown et al., 2013). Due to the short time of the mentioned medical alliance, the health technology sharing model has not yet been systematically established, and some of the technologies may be known and used by clinicians in upper-level health facilities, with it being tough for the primary ones to acquire tacit knowledge because of the equipment. At this stage, explicit knowledge, often in form of document, text and so on, was much easier to share and dominate by virtue of its codifiability (Huang et al., 2014). On the other hand, this phenomenon might be related to the loose cooperation model of the ICS, which deserved more attention and reflection. Since tacit knowledge relying on experience, interpretation and judgment, was often private, uncodifiable and difficult to record (Noh et al., 2000; Prochaska, 2011), the sharing of tacit knowledge required knowledge providers to spend more time and effort, which needed to be driven by a strong motivation existing only in strong ties (i.e., master-apprentice transmission) (Uzzi, 1997; Hansen, 2016). However, the loose collaboration model of this sort of ICS will not be able to provide sufficient opportunities for interaction, resulting in inability to develop close and stable communication between member institutions. Even worse, there was a potential competitive relationship between members that contributed to distrust among member institutions and clinicians from different institutions (Wyatt, 2001). These had led to the inability to form strong ties within this ICS, which subsequently hindered the tacit knowledge sharing. Within ICS, the dissemination of explicit knowledge cannot fully replace the key role of tacit knowledge sharing in promoting the renewal of technical concepts and expanding the practical application of technology (Provan et al., 2013). Thus, it is of concern that the lack of tacit knowledge sharing might lead to stagnation after the diffusion of health technology to a certain extent.

### Implications and strengths of the study

These findings had some implications for promoting knowledge sharing within the ICS. Firstly, it was essential to establish a tight cooperation model between member institutions of ICS. Through the unified management of personnel appointments and finances, the ICS would become a stable community of interest with the rights and obligations of its member institutions clarified, which would benefit the development of strong ties and further promote knowledge sharing and diffusion of appropriate health technology. Secondly, various measures could be taken to extend the scope of collaboration, increase opportunities of interaction between ICS members, improve the sense of belonging and recognition of member institutions to the medical alliance, such as staff exchange, clinical skills training, regular academic salons and lectures, etc. In addition, infrastructure should be strengthened to facilitate the knowledge sharing of health technology among member institutions and individuals within the ICS, such as the establishment of web-based learning communities for anytime, anywhere learning.

Besides the implications, this study was also strengthened by some features. First, integrating social networks into knowledge sharing in healthcare is a crucial contribution. Although social network analysis is becoming popular as a method to analyze social interactions and relational aspects, there still has been not many practical applications in knowledge sharing. Thus, based on the social network theory, this study investigates the influencing factors of medical and health knowledge sharing with clinicians. Second, different from previous studies which mainly focused on the intra-organizational knowledge sharing, this study paid more attention to the clinicians’ knowledge sharing in member institutions of ICS, filling the aforementioned knowledge gap by considering both intra-organizational and extra-organizational knowledge sharing. Third, the application of social network analysis on relational data made the result prediction more robust and accurate, which also helped to visually identify the network structure and clarify the influencing factors of knowledge sharing of health technology.

### Limitations and prospective research

However, there were still some limitations. First, due to time and funding constraints, this study included a limited number of variables. Subsequent studies will add relevant variables, such as personality traits and organizational factors, to enhance the explanatory power of the study. Second, as some clinicians refused to participate in this survey, there might be some deviation between the social network developed in this study and the one in the real situation. Future studies could increase the participation rate of study subjects to completely restore the real network. Third, this study selected a certain medical alliance as a case, which would weaken the generalizability of its conclusion. So, it is recommended to include more or different types of medical alliances in future studies for extrapolating the findings to a wider population.

Although these results contribute to the practice and theory, there are some possible directions for the future study. First, future research can focus on more complicated but commonly existed social networks and knowledge sharing between individuals with different professional background, such as clinicians, hospital managers, health officials, and further exploit the characteristics of their knowledge sharing behavior, as well as how they collaborate with the other group to address the health issue concertedly. Second, future research can also concentrate on the mediating variables that affect knowledge dissemination in social networks, such as motivation and altruism, in order to build a complete and credible knowledge sharing mechanism. Finally, it is also recommended to conduct future study by means of multi-channel data collection, such as mining and processing of big data (including longitudinal and cross-sectional data) through social media, and so on (Carchiolo et al., 2015).

## Conclusion

This study extended the research scope of social network theory to the field of healthcare, further advanced knowledge about the clinicians’ intra-organizational and extra-organizational knowledge sharing of health technology in the context of an ICS, which will offer a practical basis for promoting current knowledge management and technology diffusion. By applying social network analysis on the relational data, the network structure was visually described, and tie strength, trust, cognitive proximity, and reciprocal relationships were identified as the influencing factors. As the dominant role of weak ties was demonstrated in clinicians’ knowledge sharing of health technology within the ICS, it suggested that knowledge current shared was mainly explicit knowledge, not the tacit knowledge of health technology. To develop strong ties to promote tacit knowledge sharing within the ICS, some strategies are also recommended to establish a tight cooperation model between member institutions of ICS to facilitate knowledge sharing.

## Data availability statement

The raw data supporting the conclusions of this article will be made available by the authors, without undue reservation.

## Ethics statement

Written informed consent was obtained from the individual(s) for the publication of any potentially identifiable images or data included in this article.

## Author contributions

WL, ZZ, and QD contributed to the conception and design of the study. ZZ conducted the data analyses and wrote the manuscript. WL guided the whole process and reviewed the manuscript. All authors contributed to the article and approved the submitted version.

## Funding

This research was supported by the National Natural Science Foundation of China (grant number: 71704026), the Distinguished Young Scientific Research Talents Plan in Universities of Fujian Province (grant number: 2018B030), and Technology development Fund from the Department of Education of Fujian Province (grant number: 2019L3010008). And the funders had no involvement in study design, data collection, statistical analysis, and manuscript writing.

## Conflict of interest

The authors declare that the research was conducted in the absence of any commercial or financial relationships that could be construed as a potential conflict of interest.

## Publisher’s note

All claims expressed in this article are solely those of the authors and do not necessarily represent those of their affiliated organizations, or those of the publisher, the editors and the reviewers. Any product that may be evaluated in this article, or claim that may be made by its manufacturer, is not guaranteed or endorsed by the publisher.
