# Supplementary material for: Knowledge sharing of health technology among clinicians in integrated care system: The role of social networks
Source: Front Psychol. 2022 Sep 27;13:926736. doi: 10.3389/fpsyg.2022.926736 (PMC9553305; doi:10.3389/fpsyg.2022.926736)
Supplement: Supplementary file 1 [file Data_Sheet_1.doc]

**The questionnaire on the social network for knowledge sharing of health technology**

**Part 1. Social Network Status**

Notes: Review the people with whom you have shared knowledge of health technology in the past year and select the three most important people, identifying the first, second and third people in descending order of importance (if you think there are less than three people who have had an important impact, then fill in 1-3 people as appropriate. (Please include their real names due to the need of social network analysis). Answer all questions from top to bottom based on the details of the first person; then from top to bottom based on the details of the second and third person respectively.

|  | **First people**  **|______|** | **Second people**  **|______|** | **Third people**  **|______|** |
| --- | --- | --- | --- |
| 1.1 What is the direction of knowledge sharing of health technology between you and him/her?  A. He/she share knowledge to me  B. I share knowledge to him/her  C. We share knowledge with each other. |  |  |  |
| 1.2 How long have you known him/her ?  A. <6 months  B. 6 months ~ 2 years  C. 2 ~ 5 years  D. 5 ~ 10 years  E. >10 years |  |  |  |
| 1.3 In the past year, how often did you interact with him/her？  A. Less than once a year or once a year  B. 2-5 times a year  C. 6-11 times a year  D. 1-3 times a month  E. once a week and above |  |  |  |
| 1.4 What do you think is the importance of the relationship with him/her?  A. Not important  B. Normal  C. Important  D. Very important  E. Absolutely important |  |  |  |
| 1.5 Your and his/her belief in health technology  A. Different  B. Somewhat similar  C. Nearly the same  D. Almost the same |  |  |  |
| 1.6 When you encounter difficulties at work (such as doctor-patient conflicts, etc.) or feel uncomfortable, and need emotional and psychological comfort, what is the direction of emotional support between you and him/her?  A. He/she will support me  B. I will support him/her  C. We support each other  D. We do not support each other |  |  |  |
| 1.7 When you need to use things you don't have (such as medical equipment, presentation PPT, etc.) because of your work, what is the direction of your material support behavior with him/her?  A. He/she will support me  B. I will support him/her  C. We support each other  D. We do not support each other |  |  |  |

**Part 2. Information Card**

Notes: The following questions are some basic information about you and your nominee, please circle the number or fill in the blank that best matches your real situation.

|  | **First people**  **|______|** | **Second people**  **|______|** | **Third people**  **|______|** |
| --- | --- | --- | --- |
| 2.1 His/her gender  A. Male  B. Female |  |  |  |
| 2.2 His/her professional title  A. Junior  B. Intermediate  C. Deputy senior  D. Senior |  |  |  |
| 2.3 His/her department: ________ |  |  |  |
|  |  |  |  |
| 2.4 Your gender  A. Male  B. Female | | | |
| 2.5 Your professional title  A. Junior  B. Intermediate  C. Deputy senior  D. Senior | | | |
| 2.6 Your department: ________ | | | |
